# Macromolecular Proton Fraction as a Myelin Biomarker: Principles, Validation, and Applications

**DOI:** 10.3389/fnins.2022.819912

**Published:** 2022-02-09

**Authors:** Alena A. Kisel, Anna V. Naumova, Vasily L. Yarnykh

**Affiliations:** ^1^Department of Radiology, University of Washington, Seattle, WA, United States; ^2^Laboratory of Neurobiology, Tomsk State University, Tomsk, Russia

**Keywords:** macromolecular proton fraction (MPF), myelin, magnetization transfer (MT), central nervous system, brain, spinal cord, MRI, quantitative imaging

## Abstract

Macromolecular proton fraction (MPF) is a quantitative MRI parameter describing the magnetization transfer (MT) effect and defined as a relative amount of protons bound to biological macromolecules with restricted molecular motion, which participate in magnetic cross-relaxation with water protons. MPF attracted significant interest during past decade as a biomarker of myelin. The purpose of this mini review is to provide a brief but comprehensive summary of MPF mapping methods, histological validation studies, and MPF applications in neuroscience. Technically, MPF maps can be obtained using a variety of quantitative MT methods. Some of them enable clinically reasonable scan time and resolution. Recent studies demonstrated the feasibility of MPF mapping using standard clinical MRI pulse sequences, thus substantially enhancing the method availability. A number of studies in animal models demonstrated strong correlations between MPF and histological markers of myelin with a minor influence of potential confounders. Histological studies validated the capability of MPF to monitor both demyelination and re-myelination. Clinical applications of MPF have been mainly focused on multiple sclerosis where this method provided new insights into both white and gray matter pathology. Besides, several studies used MPF to investigate myelin role in other neurological and psychiatric conditions. Another promising area of MPF applications is the brain development studies. MPF demonstrated the capabilities to quantitatively characterize the earliest stage of myelination during prenatal brain maturation and protracted myelin development in adolescence. In summary, MPF mapping provides a technically mature and comprehensively validated myelin imaging technology for various preclinical and clinical neuroscience applications.

## Introduction

Investigation of myelin damage, repair, and development in the central nervous system (CNS) for the understanding of pathological mechanisms and treatment monitoring in various neurological and psychiatric conditions using non-invasive imaging methods attracted substantial attention over past two decades (Heath et al., 2018; Piredda et al., 2021). Myelin has been recognized as a key source of brain tissue contrast in MRI due to its strong effect on the nuclear magnetic resonance relaxation times T_1_ and T_2_ (Piredda et al., 2021). However, conventional MRI does not allow quantitation of the myelin content changes and lacks specificity to myelin. Recent progress in quantitative MRI methods resulted in the development of several specialized techniques with improved specificity to myelin, which potentially can be used as sources of myelin biomarkers. The underlying biophysical tissue properties affected by myelin include single- or multi-component relaxation, magnetization transfer, anisotropic diffusion, and magnetic susceptibility (Heath et al., 2018; Piredda et al., 2021). Extensive overview of the current myelin imaging methods can be found in recent reviews (Heath et al., 2018; Piredda et al., 2021). Several recently published meta-analyses (Mancini et al., 2020; Lazari and Lipp, 2021; van der Weijden et al., 2021) compared multiple histological validation studies of prospective myelin imaging biomarkers. While there is no evidence of a superiority of any single myelin imaging method to date, the above studies consistently identified the macromolecular proton fraction (MPF) among the parameters enabling the strongest correlations with myelin histology.

MPF is a parameter describing the magnetization transfer (MT) effect and defined as a relative amount of protons bound to biological macromolecules with restricted molecular motion, which participate in magnetic cross-relaxation with free water protons (Yarnykh, 2012). MPF offers several important advantages as a myelin biomarker. Particularly, MPF has fewer physiological confounders as compared to myelin measures based on diffusion, relaxation, and susceptibility. Diffusion indexes associated with myelination, such as radial diffusivity and fractional anisotropy (Song et al., 2002), are affected by microstructural tissue organization and spatial orientation of myelinated fibers (Wheeler-Kingshott and Cercignani, 2009), whereas MPF is independent of these factors (Underhill et al., 2009; Stikov et al., 2011). Relaxation times T_1_, T_2_, and T_2_* and magnetic susceptibility in neural tissues are mainly determined by both myelination and concentration of iron (Stüber et al., 2014; Duyn and Schenck, 2017). As a consequence, a popular myelin biomarker, myelin water fraction measured from multi-component relaxation analysis (MacKay et al., 1994; Deoni et al., 2008; Hwang et al., 2010) is also influenced by the iron content (Birkl et al., 2019). In contrast, MPF is not affected by iron or other paramagnetic ions in tissues (Li et al., 2016; Trujillo et al., 2017a; Yarnykh et al., 2018a). MPF is also independent of magnetic field strength. *In vivo* MPF measurements in the brain white matter (WM) and gray matter (GM) appeared quantitatively consistent in a wide range of magnetic fields from 0.5 T (Anisimov et al., 2020) to 11.7 T (Naumova et al., 2017). Therefore, MPF provides an attractive approach as a uniform quantitative scale of myelin measurements across a variety of human and animal MRI platforms. Finally, MPF maps can be obtained using routine MRI equipment without modification of original manufacturers’ pulse sequences (Yarnykh et al., 2018b; Korostyshevskaya et al., 2019; Smirnova et al., 2021) thus facilitating clinical translation of this technology.

While MPF mapping has been in use for almost 20 years, the current literature lacks a review specifically focused on MPF as a myelin biomarker. In this review, we sought to provide a brief but comprehensive summary of existing MPF mapping techniques, histological validation studies, and MPF applications in neuroscience. The review is based on PubMed literature search including synonyms of MPF (such as “bound pool fraction,” “bound proton fraction,” “semisolid pool fraction,” “semisolid proton fraction,” and “bound water fraction”) and a similar quantity, pool size ratio (PSR) (Gochberg and Gore, 2003) related to MPF as PSR = MPF/(1−MPF). The term MPF is uniformly used below, although different notations can be found in original publications. The search methodology is detailed in Supplementary Material.

## Literature Review

### Macromolecular Proton Fraction Mapping Methods

The group of methods allowing reconstruction of MPF maps alone or in combination with other MT parametric maps is commonly referred to as quantitative MT (qMT). The two-pool model of MT (Morrison and Henkelman, 1995) provides a general theoretical framework for all qMT techniques. Within this model, tissue is represented by two proton magnetization reservoirs (free water and macromolecular pool), where the process of magnetization exchange is described by the cross-relaxation rate constant and MPF. Each pool has own transverse and longitudinal relaxation times. We recommend reviews (Henkelman et al., 2001; Sled, 2018) for more details of biophysics of the MT effect. The model parameters can be estimated using the two basic strategies: analysis of a signal behavior in response to off-resonance radiofrequency (RF) saturation with variable offset frequency and power (Z-spectroscopy) and analysis of temporal signal evolution after initial semi-selective perturbation of either water or macromolecular magnetization, which is described by the bi-exponential function (cross-relaxometry). In the contemporary formulation, the two-pool model for tissues includes the super Lorentzian spectral line-shape of the macromolecular pool and Lorentzian line-shape of the free water pool (Morrison and Henkelman, 1995). This model was adapted to the pulsed steady-state saturation regimen, which are achieved using MRI pulse sequences, and enabled the first experimental demonstrations of MPF maps of the human brain along with the maps of other model parameters using multi-parameter voxel-wise fit of Z-spectral images (Sled and Pike, 2001; Yarnykh, 2002). Also, several methods based on the analysis of bi-exponential longitudinal relaxation were reported. The RF pulse excitation schemes in these techniques may vary and may be preferentially targeted at either the free water or macromolecular protons. Within this paradigm, the techniques based on selective inversion of free water magnetization (Gochberg and Gore, 2003, 2007; Dortch et al., 2011; Cronin et al., 2020), stimulated echo preparation (Ropele et al., 2003; Soellinger et al., 2011), and broadband saturation of the macromolecular pool (van Gelderen et al., 2017) were developed. On-resonance saturation of water protons caused by readout RF pulses in fast gradient-echo sequences also can be used as a tool for MPF mapping (Gloor et al., 2008; Garcia et al., 2010; Bayer et al., 2021) based on steady-state models, where the signal is sampled as a function of the excitation pulse duration and flip angle.

The two-pool model contains six independent parameters. None of existing qMT methods can simultaneously measure all of them, and certain assumptions are required. In Z-spectroscopic methods, T_1_ of the pools are unavailable from the model fit and are mathematically coupled with cross-relaxation parameters. Accordingly, separate T_1_ mapping is usually performed, and T_1_ of the free water pool (T_1_^F^) is calculated under some assumption about T_1_ of the macromolecular pool (T_1_^M^). The common assumptions include equating them (Yarnykh, 2002) or setting T_1_^M^ = 1 s (Morrison and Henkelman, 1995). T_1_ measurements also may need to be corrected for the cross-relaxation effect (Mossahebi et al., 2014). In cross-relaxometric experiments, T_2_ of the pools cannot be extracted from data and are usually estimated by simulations in order to approximate the initial magnetization state (Gochberg and Gore, 2003; Ropele et al., 2003; Gloor et al., 2008; van Gelderen et al., 2017).

Simultaneous estimation of many parameters (usually 4 or 3) in qMT techniques requires a large number of source images, which result in impractically long data acquisition (>30 min for the human whole-brain examination), particularly in earlier methods (Sled and Pike, 2001; Ramani et al., 2002; Yarnykh, 2002; Gloor et al., 2008; Dortch et al., 2011). Additional B_0_ and B_1_ maps are also frequently used for correction of errors caused by field non-uniformities (Sled and Pike, 2001; Gloor et al., 2008; Yarnykh, 2012; Boudreau et al., 2018), thus further increasing examination time. Reduction of the acquisition time can be achieved using several strategies including optimized schedules of variable experimental parameters (Cercignani and Alexander, 2006; Li et al., 2010; Levesque et al., 2011; Battiston et al., 2018; Boudreau and Pike, 2018; Cronin et al., 2020), specialized sequences enabling acquisition of several data points within a single scan (Soellinger et al., 2011; van Gelderen et al., 2017; Battiston et al., 2019), and a reduction of the model dimension by constraining certain parameters or their combinations (Ropele et al., 2003; Yarnykh and Yuan, 2004; Cercignani et al., 2005; Yarnykh, 2012). The last approach resulted in the most radical solution providing MPF estimation from a single spoiled gradient-echo image with off-resonance saturation (Yarnykh, 2012) and a T_1_ map calculated using the two-point variable flip angle method (Deoni et al., 2005). The single-point method exploits negligible variability of the cross-relaxation rate constant (in the macromolecules-to-water direction), T_2_ of macromolecular protons, and the product of observed R_1_ = 1/T_1_ and T_2_ of the free water pool in brain tissues (Yarnykh, 2012). These quantities are fixed in the reconstruction algorithm, thus making MPF the only adjustable parameter. Further acceleration of the single-point technique included elimination of a reference image (Yarnykh, 2016), which is usually needed in most qMT techniques, exclusion of B_0_ mapping due to a negligible effect of B_0_-related errors (Yarnykh et al., 2020), and a new data-driven algorithm for B_1_ non-uniformity correction (Yarnykh, 2021), which obviates commonly used in qMT protocols B_1_ mapping sequences. With these improvements, the entire single-point MPF mapping protocol consists of only three spoiled gradient-echo sequences providing MT-, T_1_-, and proton-density-weighted images. Acceleration achieved with the single-point MPF mapping method can be converted into either high-resolution acquisition with a generally acceptable for human neuroscience applications scan time (about 15 min for a whole-brain dataset with isotropic 1.25 mm^3^ resolution; Yarnykh, 2021) or fast clinically targeted protocols (3.5 min for a whole-brain dataset with 1.5 × 1.5 × 5.0 mm^3^ resolution; Yarnykh et al., 2018b). Due to lesser sensitivity to noise, single-point MPF mapping showed improved reproducibility compared to multi-parameter techniques. Particularly, reported coefficients of variation of repeated measurements in the human brain were 1–2% for the single-point method (Yarnykh et al., 2020; Smirnova et al., 2021) and about 5% for multi-parameter qMT (Davies et al., 2004; Levesque et al., 2010b).

Software availability is an important aspect of future MPF mapping applications. While most studies to date utilized custom-written software tools, we identified two open-source freely available software packages enabling MPF map reconstruction. Quantitative MRI analysis MATLAB (MathWorks, Inc.; Natick, MA, United States) library “qMRLab” (Karakuzu et al., 2020; qMRLab, 2021) allows MPF map reconstruction based on several widely used multi-parameter qMT fit models (Sled and Pike, 2001; Gloor et al., 2008; Li et al., 2010). Specifically targeted at the single-point method (Yarnykh, 2012, 2016) C++ language software “MPF_map” is available from the website (Macromolecular Proton Fraction [MPF], 2022).

### Validation in Animal Models

Macromolecular proton fraction measurements were compared with histological myelin assessment in a variety of animal models including normal animal brain (Underhill et al., 2011; Khodanovich et al., 2017) and spinal cord (Dula et al., 2010), cuprizone-induced demyelination in mice (Thiessen et al., 2013; Turati et al., 2015; Khodanovich et al., 2017, 2019; Soustelle et al., 2019, 2021), experimental autoimmune encephalomyelitis in rats (Rausch et al., 2009), lipopolysaccharide-induced focal demyelination in rats (Janve et al., 2013), hexachlorophene-induced intramyelinic edema in rats (Harkins et al., 2013), genetic hypomyelination (Ou et al., 2009a,b; West et al., 2018) and hypermyelination (West et al., 2018) in mice, ischemic stroke in rats (Khodanovich et al., 2018, 2021), spinal cord injury in primates (Wang et al., 2016, 2019; Wu et al., 2020), and demyelinated peripheral nerve *ex vivo* (Odrobina et al., 2005; Ou et al., 2009a). All studies reported qualitative correspondence of demyelinated anatomical zones with a reduced MPF and/or MPF reduction associated with demyelination relative to a control sample. When histology was quantitatively assessed, MPF demonstrated strong correlations with histological myelin markers characterized by correlation coefficients in a range 0.7–0.99 (Underhill et al., 2011; Janve et al., 2013; Thiessen et al., 2013; Turati et al., 2015; Khodanovich et al., 2017, 2018, 2019, 2021; West et al., 2018; Soustelle et al., 2019, 2021).

Several works focused on validation of MPF as a tool for monitoring re-myelination that is of critical importance for therapeutic intervention studies. An increase in MPF was correlated with re-myelination after withdrawal of cuprizone (Turati et al., 2015; Khodanovich et al., 2019) and replicated restoration of oligodendrogenesis (Khodanovich et al., 2019) in WM and GM of cuprizone-pretreated mice. In the stroke model, MPF showed a unique capability to identify local post-ischemic remyelination, which was unobservable with conventional imaging techniques (Khodanovich et al., 2021).

Animal studies provided important insights into specificity of MPF to myelin and a role of potential confounders. MPF in normal brain tissues is largely independent of the total cell count and axonal density (Underhill et al., 2011). The loss of axons and neurons in ischemic stroke did not affect MPF (Khodanovich et al., 2018). MPF in the ischemic infarct was also found to be insensitive to microglial (Khodanovich et al., 2018) and astroglial (Khodanovich et al., 2021) proliferation, which represent pathological hallmarks of sub-acute and chronic stroke lesions. At the same time, due to dilution of the macromolecular content, MPF is affected by edema (Stanisz et al., 2004; Harkins et al., 2013; Khodanovich et al., 2018), which may cause up to 10–15% overestimation of myelin loss by MPF in acute stroke (Khodanovich et al., 2018). Multi-modal approaches were proposed to correct the effect of water content changes on MPF, particularly using proton density (Giacomini et al., 2009; Mossahebi et al., 2015) or T_2_ measurements (Khodanovich et al., 2018), but they need more rigorous validation.

Animal models of brain development were not studied as extensively as demyelination models. Nevertheless, several publications indicate utility of MPF for monitoring normal or abnormal myelin development (Samsonov et al., 2012; Lu et al., 2018; Goussakov et al., 2019). Using MPF, these studies demonstrated dramatic distinctions in temporal myelination trajectories between the genetic canine demyelination model and normal animals (Samsonov et al., 2012), widespread effect of ischemia-hypoxia on postnatal myelination in murine WM and GM (Goussakov et al., 2019), and alterations in age-dependent myelin development caused by microbiota in mice (Lu et al., 2018).

### Neuroscience Applications

The most common primary demyelinating disease, multiple sclerosis (MS) attracted significant interest as an area of clinical MPF applications. The earliest technical development studies (Sled and Pike, 2001; Yarnykh, 2002) demonstrated that MPF maps clearly depict MS lesions in WM as areas of low MPF. Subsequent reports identified the capability of MPF to detect microscopic demyelination in normal-appearing WM (NAWM) (Davies et al., 2003, 2004; Tozer et al., 2003, 2005; Narayanan et al., 2006; Cercignani et al., 2009; Spano et al., 2010; Yarnykh et al., 2015, 2018a; Bagnato et al., 2020). However, some studies did not find significant NAWM MPF differences between patients and controls (Bagnato et al., 2018; McKeithan et al., 2019), probably due to methodological distinctions in acquisition protocols. MPF provided new insights into lesion pathology in MS enabling studies of demyelination heterogeneity (Levesque et al., 2005; Clarke et al., 2021) and temporal evolution (Giacomini et al., 2009; Levesque et al., 2010a). Post-mortem MPF and histology studies of MS (Schmierer et al., 2007; Bagnato et al., 2018) confirmed good agreement between demyelination and a reduced MPF. The majority of MS studies utilized multipoint techniques with either off-resonance saturation (Sled and Pike, 2001; Yarnykh, 2002; Tozer et al., 2003, 2005; Davies et al., 2003, 2004; Levesque et al., 2005; Narayanan et al., 2006; Schmierer et al., 2007; Cercignani et al., 2009; Giacomini et al., 2009; Levesque et al., 2010a; Spano et al., 2010) or selective inversion-recovery preparation (Bagnato et al., 2018, 2020; McKeithan et al., 2019; Clarke et al., 2021) to obtain MPF maps. The single-point method (Yarnykh, 2012) extended the area of MPF applications to GM (Yarnykh et al., 2015, 2018a) and demonstrated strong associations of GM MPF with MS disability scales and disease phenotype (Yarnykh et al., 2015). The single-point method was also adapted to the spinal cord imaging (Smith et al., 2014) and showed a significant reduction of both NAWM and GM MPF in MS (Smith et al., 2017).

Applications of MPF in other conditions are scarce. The summary of non-MS clinical applications of MPF and key findings is provided in Table 1. Collectively, these studies indicate growing usage of MPF as an exploratory myelin imaging tool in diseases not primarily related to myelin pathology and suggest that MPF mapping adds a new dimension in quantitative clinical neuroimaging.

**TABLE 1:** Summary of MPF applications in human conditions other than multiple sclerosis (MS).

Condition	Measurement technique	Main findings
Alzheimer’s disease (AD)	Multipoint off-resonance (Ridha et al., 2007; Kiefer et al., 2009; Giulietti et al., 2012).	Decreased hippocampal combined index MPF/[(1 - MPF)R_1_] (Ridha et al., 2007). Increased hippocampal MPF (Kiefer et al., 2009). No significant effect on MPF according to voxel-based analysis but a reduced forward exchange rate constant in multiple cortical regions (Giulietti et al., 2012).
Genetic risk variants of AD	Multipoint off-resonance (Mole et al., 2020a,b).	Reduced MPF in the right parahippocampal cingulum (Mole et al., 2020a) and left thalamus (Mole et al., 2020b) in participants with APOE-ε4 genetic risk factor and family history of AD.
Interferon-α induced fatigue	Steady-state multipoint on-resonance (Dowell et al., 2016).	No significant effect on MPF according to voxel-based analysis but an increased forward exchange rate constant in the striatum and insula (Dowell et al., 2016).
Huntington’s disease	Multipoint off-resonance (Bourbon-Teles et al., 2019; Casella et al., 2020).	MPF decrease in whole-brain WM (Bourbon-Teles et al., 2019; Casella et al., 2020). Motor training induced a significant MPF increase in the corpus callosum and motor pathways (Casella et al., 2020).
Mild traumatic brain injury	Single-point (Petrie et al., 2014).	Significant MPF reduction in whole-brain WM and GM (Petrie et al., 2014).
Normal aging	Multipoint off-resonance (Metzler-Baddeley et al., 2019b; Coad et al., 2020; Mole et al., 2020a).	Significant negative correlations between MPF and age in the fornix (Metzler-Baddeley et al., 2019b; Coad et al., 2020) and whole-brain WM (Mole et al., 2020a).
Obesity	Multipoint off-resonance (Metzler-Baddeley et al., 2019a).	MPF in the fornix negatively correlated with markers of obesity (Metzler-Baddeley et al., 2019a).
Brain tumors	Multipoint off-resonance (Yarnykh, 2002; Tozer et al., 2011; Mehrabian et al., 2018a,b); steady-state multipoint on-resonance (Garcia et al., 2015); single-point (Korostyshevskaya et al., 2018).	Variable MPF decrease relative to normal WM in all studied tumors including gliomas (Yarnykh, 2002; Tozer et al., 2011; Garcia et al., 2015; Mehrabian et al., 2018a,b), meningiomas (Garcia et al., 2015), and brain metastases (Garcia et al., 2015). Increased MPF relative to fetal brain tissue in fetal collagen-rich medulloblastoma (Korostyshevskaya et al., 2018).
Parkinson’s disease	Single-point and multipoint off-resonance (Trujillo et al., 2017b).	Increased MPF in the substantia nigra, good agreement between single- and multi-point techniques (Trujillo et al., 2017b).
Adrenomyeloneuropathy	Multipoint off-resonance (Smith et al., 2009).	Significantly decreased MPF in the dorsal column of the spinal cord with no differences in the lateral columns and GM (Smith et al., 2009).
Fabry disease	Multipoint off-resonance (Underhill et al., 2015).	MPF reduction in left posterior brain WM, which was negatively associated with age (Underhill et al., 2015).
Myotonic dystrophy type 1	Multipoint off-resonance (Leddy et al., 2021).	Reduced MPF in WM lesions, no differences between patients and controls in NAWM (Leddy et al., 2021).
Schizophrenia	Multipoint off-resonance (Kiefer et al., 2004; Kalus et al., 2005); single-point (Smirnova et al., 2021; Sui et al., 2021).	No significant effect on MPF in the hippocampus (Kiefer et al., 2004) and amygdala (Kalus et al., 2005). Significant MPF decrease in whole-brain WM and GM associated with negative symptoms. Significant negative correlation between MPF in WM and disease duration (Smirnova et al., 2021). Voxel-based patterns of variable increase and decrease in cortical MPF depending on the disease duration. Geometric non-linearity of the cortical MPF profile decreased in patients and negatively correlated with disease duration (Sui et al., 2021).
Small vessel disease (white matter hyperintensities)	Non-conventional estimation as MT ratio/T_1_ (Iordanishvili et al., 2019).	Decreased MPF in WM hyperintensities. Periventricular hyperintensities had lower MPF than deep WM ones. A decrease in MPF corresponds to lesion severity according to Fazekas scale (Iordanishvili et al., 2019).
Systemic inflammation	Steady-state multipoint on-resonance (Harrison et al., 2015).	No significant effect on MPF according to voxel-based analysis but an increased forward exchange rate constant in the insula (Harrison et al., 2015).

**FIGURE 1:**
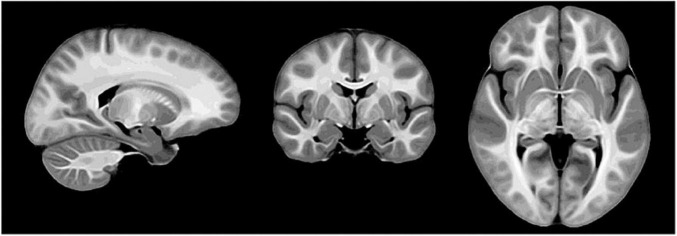
Study-specific template derived from macromolecular proton fraction (MPF) maps of 146 adolescent study subjects (reprinted from Corrigan et al. (2021); free PMC article).

Brain development is another promising area of MPF applications since myelination is a fundamental component of CNS maturation. Fast single-point MPF mapping was used to investigate the earliest stage of myelin development in the fetal brain and showed close correlations with gestational age in the anatomic regions with known prenatal myelination onset (Yarnykh et al., 2018b; Korostyshevskaya et al., 2019). It was also demonstrated that the single-point method enables reliable measurements of very low MPF values in the fetal brain, which are about fivefold lower than MPF in adult WM (Yarnykh et al., 2018b; Korostyshevskaya et al., 2018, 2019). A recent large-scale study investigated spatiotemporal trajectories of protracted myelin development during adolescence using high-resolution MPF maps and identified that GM myelination is characterized by a significantly faster rate as compared to WM and correlates with puberty (Corrigan et al., 2021). In the technical aspect, MPF maps used in this study demonstrated unprecedented anatomical contrast, as illustrated by the study template in Figure 1.

## Discussion

Substantial body of evidence confirms high sensitivity and specificity of MPF to myelin. At the same time, early brain development (pre- or postnatal) remains an area where animal model studies could provide an important background for future clinical applications. Water content alterations remain a sole major confounder of MPF according to prior studies. Development of multimodal imaging approaches to mitigate the effect of water content changes would be of crucial value for MPF application in pathological conditions involving significant edema component, such as acute stroke or brain injury. In the perspective of clinical translation, MPF mapping should enable sufficiently fast acquisition and independence of a particular imaging platform. The last requirement can be met, if a technique employs standard pulse sequences provided by most MRI equipment manufacturers. In the current state of development, only two approaches [single-point synthetic-reference method (Yarnykh, 2016) and selective inversion-recovery with optimized sampling and accelerated acquisition (Cronin et al., 2020)] allow designs of MPF mapping protocols based on unmodified sequences and provide whole-brain acquisition in less than 10 min. Since fast MPF mapping employs constrained reconstruction algorithms, consensus is needed regarding the details of the fit procedure and values of constrained parameters to facilitate comparisons between multiple studies. This aspect may involve further model refinements, such as more accurate parameters modeling macromolecular protons (Helms and Hagberg, 2009; van Gelderen et al., 2016, 2017). Finally, multi-platform protocol harmonization and repeatability studies are needed to enable MPF applications in multicenter clinical trials.

## Author Contributions

AK and AN contributed to the literature research, and manuscript drafting and formatting according to the journal guidelines. VY overviewed the general concept and design of the study and made final corrections to the manuscript. All authors contributed to the article and approved the submitted version.

## Conflict of Interest

The authors declare that the research was conducted in the absence of any commercial or financial relationships that could be construed as a potential conflict of interest.

## Publisher’s Note

All claims expressed in this article are solely those of the authors and do not necessarily represent those of their affiliated organizations, or those of the publisher, the editors and the reviewers. Any product that may be evaluated in this article, or claim that may be made by its manufacturer, is not guaranteed or endorsed by the publisher.
