# Supplementary material for: Macromolecular Proton Fraction as a Myelin Biomarker: Principles, Validation, and Applications
Source: Front Neurosci. 2022 Feb 9;16:819912. doi: 10.3389/fnins.2022.819912 (PMC8863973; doi:10.3389/fnins.2022.819912)
Supplement: Supplementary file 1 [file Data_Sheet_1.PDF]

Supplementary Material

1 Literature Search Methodology

PubMed literature search was performed taking into account the following terminological considerations. Historically, different terminology has been used by different groups in the definition of a theoretical model of the MT effect. The model itself is commonly referred to as the “two-pool model” or “binary spin-bath model” (Morrison and Henkelman, 1995; Sled and Pike, 2001). Several synonyms of the same physical quantity of interest (MPF) can be found in the literature including “macromolecular proton fraction” (Davies et al., 2003), “bound pool fraction” (Lee and Dagher, 1997), “bound proton fraction” (Tozer at al., 2003), "semisolid pool fraction" (Stanisz et al., 2004), “semisolid proton fraction” (Yarnykh, 2002), and “bound water fraction” (Ramani et al., 2002). A slightly different definition of the macromolecular proton content in the MT model termed “pool size ratio (PSR)” (Gochberg and Gore, 2003) or “fractional size of the restricted pool” (Sled and Pike, 2001) also has been used. MPF and PSR have a simple relationship: PSR=MPF/(1-MPF). From the mathematical standpoint, these definitions of the macromolecular proton content are related to the normalization of equilibrium magnetization to the molar concentration of either all protons in tissue or only the mobile water protons (the source of MRI-detectable signal). In the context of pathological changes, both MPF and PSR are equivalent, though their absolute values may differ. In view of these considerations, we uniformly use the term MPF below, although different notations could be found in the original publications. The quantitative imaging techniques for MPF mapping also have different original notations referring to either MT or cross-relaxation (Yarnykh 2002, Yarnykh and Yuan, 2004). We further attempted to limit search to the studies of CNS or myelin. The final PubMed search query was as follows:

Search: ("macromolecular proton fraction" OR "bound pool fraction" OR "bound proton fraction" OR "pool size ratio" OR "semisolid proton fraction" OR "bound water fraction" OR "quantitative magnetization transfer" OR "quantitative MT" OR "qMT" OR "cross-relaxation" OR "semisolid pool" OR "bound pool" OR "two-pool model" OR "binary spin-bath model") AND (myelin OR brain OR "spinal cord" OR "central nervous system" OR demyelination) AND ("magnetic resonance imaging" OR "MRI" OR "magnetic resonance") Filters: from 2001/1/1 - 3000/12/12 Sort by: Publication Date

The time interval was limited to 2001, the year when the first in vivo MPF map was published (Sled and Pike, 2001).

The search resulted in 187 references, which were manually checked for the correspondence to the review topic. References were excluded if there were no reported measurements of MPF or a similar quantity, parametric maps were not reconstructed, or the study was not focused on the brain or spinal cord. The refined set of references included 109 full text articles. Additional references to non-imaging studies were added according to their methodological relevance.
